# Cardiovascular Comorbidities Relate More than Others with Disease Activity in Rheumatoid Arthritis

**DOI:** 10.1371/journal.pone.0146991

**Published:** 2016-01-12

**Authors:** Gloria Crepaldi, Carlo Alberto Scirè, Greta Carrara, Garifallia Sakellariou, Roberto Caporali, Ihsane Hmamouchi, Maxime Dougados, Carlomaurizio Montecucco

**Affiliations:** 1 Department of Rheumatology, IRCCS Policlinico San Matteo Foundation, Pavia, Italy; 2 Epidemiology Unit, Italian Society for Rheumatology, Milan, Italy; 3 Mohammed V Souissi University, Faculty of Medicine, Laboratory of Biostatistics, Clinical Research and Epidemiology (LBRCE), Rabat, Morocco; 4 Rheumatology B Department, Hospital Cochin, Medicine Faculty, Paris-Descartes University, Paris, France; VU University Medical Center, NETHERLANDS

## Abstract

**Objectives:**

To explore the influence of comorbidities on clinical outcomes and disease activity in rheumatoid arthritis (RA).

**Methods:**

In patients included in the cross-sectional observational multicenter international study COMORA, demographics, disease characteristics and comorbidities (hypertension, diabetes, hyperlipidemia, renal failure, ischemic heart disease, stroke, cancer, gastro-intestinal ulcers, hepatitis, depression, chronic pulmonary disease, obesity) were collected. Multivariable linear regression models explored the relationship between each comorbidity and disease activity measures: 28-swollen joint count (SJC), 28-tender joint count (TJC), erythrocyte sedimentation rate (ESR), patient’s and physician’s global assessment (PtGA, PhGA), patient reported fatigue and 28-Disease Activity Score (DAS28). Results are expressed as mean difference (MD) adjusted for the main confounders (age, gender, disease characteristics and treatment).

**Results:**

A total of 3,920 patients were included: age (mean ±SD) 56.27 ±13.03 yrs, female 81.65%, disease duration median 7.08 yrs (IQR 2.97–13.27), DAS28 (mean ±SD) 3.74 ± 1.55. Patients with diabetes had more swollen and tender joints and worse PtGA and PhGA (MD +1.06, +0.93, +0.53 and +0.54, respectively). Patients with hyperlipidemia had a lower number of swollen and tender joints, lower ESR and better PtGA and PhGA (MD -0.77, -0.56, -3.56, -0.31 and -0.35, respectively). Patients with history of ischemic heart disease and obese patients had more tender joints (MD +1.27 and +1.07) and higher ESR levels (MD +5.64 and +5.20). DAS28 is influenced exclusively by cardiovascular comorbidities, in particular diabetes, hyperlipidemia, ischemic heart disease and obesity.

**Conclusions:**

Cardiovascular comorbidities relate more than others with disease activity in RA. Diabetes and hyperlipidemia in particular seem associated with higher and lower disease activity respectively influencing almost all considered outcomes, suggesting a special importance of this pattern of comorbidities in disease activity assessment and clinical management.

## Introduction

With clinical remission becoming a feasible target, the suppression of disease activity is the current goal of the treatment in rheumatoid arthritis (RA) [1]. Despite treat-to-target strategy is supported by a number of clinical trials [2], some concerns on its implementation into clinical practice have been recently raised by rheumatology opinion leaders [3], because composite indexes, used to assess disease activity in clinical practice, may be affected by comorbidities or other patient-related factors and might not truly be representative of disease activity.

In the first study reporting the influence of comorbidities on disease course in RA, about 27% of patients with early RA had at least one chronic coexisting disease, but no significant difference in disease activity score (DAS), nor in treatment, between patients with or without comorbidities, was described during the first year of treatment [4]. However in this study, the independent relationship between comorbidity and disease activity was not evaluated by multi-variable models. In a more recent long-term follow-up study the relationship between comorbidities and disease activity in RA patients has been evaluated at the time of diagnosis and after 15 years [5]. In that context the number of comorbidities increased during the follow up period and the 28 joints-Disease Activity Score (DAS28) over time was higher in groups with more comorbidities [5]. DAS calculation, in fact, includes variables, such as pain related to tender joints and the patients global health (GH) or global disease activity assessment (PtGA), showing a strong positive correlation with the number of other coexisting diseases [6,7] and reflecting painful conditions other than RA [8]. Ranganath et al. evaluated the association between comorbidities and disease activity assessment in a large subcohort of 1548 RA outpatients in the CORRONA registry. The results of the multivariate analysis showed that the number of comorbidities affected directly the outcome measures and disease activity [9].

An increasing number of comorbidities leads also to a decrease of health-related quality of life (HRQoL) in RA patients [10] and therefore a multimorbidity index based on HRQoL was recently developed to be applied in clinical trials and epidemiological studies in cohort of patients with chronic conditions such as RA [11].

However, not only the number but also the type of comorbidity is relevant in the setting of a treat-to-target strategy. The association between RA and cardiovascular comorbidities in particular is well recognized [12] and the increased incidence of cardiovascular events in RA patients has a demonstrated strength association with disease activity [13].

The relationship between specific comorbidities and single outcomes of disease activity has not been fully investigated yet. To be able to dissect the relative influence of different comorbidities on disease activity and disease activity metrics would increase our knowledge and improve feasibility of the implementation of a treat-to-target strategy in clinical practice.

The present study has therefore two major objectives: to evaluate, in a large sample of patients with RA, enrolled in the international, cross-sectional study COMORA [14], which comorbidities may influence each component of the clinical composite measures used to assess disease activity and to identify their effect on DAS28.

## Patients and Methods

### Study population

In the cross-sectional observational multicenter international study COMORA, consecutive patients visiting the participating rheumatologist were enrolled if they were at least 18 years old, fulfilled the 1987 American College of Rheumatology classification criteria for RA [15] and were able to understand and complete the questionnaires. COMORA study original protocol was reviewed and approved by the Bioethics committee of the Istituto di Ricovero e Cura a Carattere Scientifico Policlinico San Matteo Foundation of Pavia for Italy (approval number 759/D.G. 15/07/2011). All participants provided their written informed consent before enrolment. Data were then collected with a specifically created case report form during a single outpatient visit. Age, gender, body mass index, smoking status, alcohol intake, marital status and level of education were recorded. Disease activity was assessed using DAS28 with erythrocyte sedimentation rate (ESR). Measures of disease severity (disease duration, serology and structural damage) and eventual extra-articular manifestation of disease were also recorded. Data about past and ongoing therapy were collected including non-steroidal anti-inflammatory drugs, corticosteroids, conventional and biological disease modifying anti-rheumatic drugs (DMARDs). Patients were questioned about history or current evidence of comorbidities, coexisting risk factors for cardiovascular diseases, infectious disease and cancer and compliance with current national recommendation regarding management of comorbidities. Detailed description about data collection and analysis has been previously reported in the COMORA main study [14].

### Study variables

The relationship with disease activity measures was evaluated for the following comorbidities: hypertension, diabetes mellitus, hyperlipidemia, renal deficiency, history of ischemic heart disease or stroke, cancer (colon, skin, lung, breast, uterus, prostate), gastro-intestinal ulcers, viral hepatitis, depression, chronic pulmonary disease and obesity (BMI ≥ 30 kg/m^2^). The presence of these comorbidities in the clinical history (yes/no) was evaluated through a face-to-face interview at a dedicated study visit and through review of the medical record.

Clinical and laboratory measures of disease activity were considered as outcome. Clinical measures of disease activity included 28-swollen joint count (SJC), 28-tender joint count (TJC), ESR, physician’s global assessment of disease (PhGA) and patient reported outcomes such as PtGA and patient reported fatigue scored on a 10 cm visual analogue scale. DAS28-ESR was also calculated.

The presence of potential confounders was also taken into account, in particular age, gender, disease duration in years, positive or negative serology for rheumatoid factor and/or anti-citrullinated protein antibodies (ACPA), smoking status and current intake of the following treatments: corticosteroids (equivalent prednisone mg/day), non-steroidal anti-inflammatory drugs (NSAIDs) and DMARDs in monotherapy or combination therapy including biologics. The current administration of statins and the cumulative dose of corticosteroids were recorded as well.

### Statistical methods

The influence of the presence of a specific comorbidity with single disease activity measures was evaluated by linear regression models, and results presented as mean difference (MD) and 95% confidence interval (CI). Given that comorbidities may influence disease activity either limiting the proper treatment because contra-indications or inflating disease activity measures for reason independent from the true disease activity, further sets of adjusted models were fitted. A first set of adjusted models was fitted for each comorbidity separately, adjusting for major confounders: demographics (age, gender), disease-related variables (disease duration, serology), and treatment variables (glucocorticoids, NSAIDs, DMARDs). A second set of adjusted models evaluated the independent association of each comorbidity with the outcomes, also including the other comorbidities as confounders. Analyses were performed using STATA software package (StataCorp, 2009, release 11, TX, USA).

## Results

### Study population

A total of 3,920 patients from 17 countries were included. The mean (standard deviation, SD) age was 56.3 (± 13.0) years, 81.7% were women, the median disease duration (interquartile range, IQR) was 7.1 (3.0–13.3) years and the mean (SD) DAS28 was 3.7 (± 1.6). The baseline patient and disease characteristics are summarized in Table 1 presented as number and percentage, mean ± SD and median (interquartile range) when appropriate. Results of the analyses of association between comorbidities and disease activity are presented below as MD, adjusted for all confounders including other comorbidities.

**Table 1:** Characteristics of the study population (N = 3920).

Socio-demographics		
Female sex—no. (%)		3191 (81.7)
Age (year)—mean ±SD		56.3 ± 13.0
BMI—mean ±SD		26.1 ± 5.5
Smoking status—no. (%)	Never	2416 (63.4)
	Cessation	895 (23.4)
	Current smoker	503 (13.2)
**Disease characteristics—Activity of the disease**		
Number of swollen joints—median (interquartile range)		1 (0–4)
Number of tender joints—median (interquartile range)		2 (0–6)
ESR (mm/Hr)—median (interquartile range)		20 (10–37)
Physician's global assessment—median (interquartile range)		3 (1–4)
DAS28—mean ±SD		3.7 ± 1.6
Disease characteristics—Severity of the disease		
Disease duration (years)—median (interquartile range)		7.1 (3.0–13.3)
Serology negative^a^ (Rheumatoid Factor AND ACPA)—no. (%)		731 (19.3)
Low positive^b^ Rheumatoid Factor OR low positive ACPA—no. (%)		980 (26.1)
High positive^c^ Rheumatoid Factor OR high positive ACPA—no. (%)		2238 (59.1)
Unequivocal radiological erosion—no. (%)		2030 (53.8)
Disease treatments		
Corticosteroids (mg/day)—mean ±SD		3.5 ± 4.6
NSAIDs—no (%)	< 1 day/week	2117 (54.5)
	1 to 5 days/week	383 (9.9)
	≥ 5 days/week	1383 (35.6)
DMARDs—no (%)	No DMARDs	275 (7.0)
	Monotherapy without MTX and Biologics	699 (17.8)
	Monotherapy with MTX	1130 (28.8)
	Combination without Biologics	834 (21.3)
	Biologics	982 (25.1)

BMI, body mass index; ESR, erythrocyte sedimentation rate; DAS28, Disease Activity Score using 28 joints; ACPA, anti-citrullinated protein antibodies; NSAIDs, non-steroidal anti-inflammatory drugs; DMARDs, disease modifying anti-rheumatic drugs; MTX, methotrexate.

^a^Serology negative = values ≤upper limit Normal (ULN) for the lab and assays

^b^Serology low positive = values >ULN and ≤3 x ULN (where RF is only available as positive or negative, a positive result should be scored “low positive”)

^c^Serology high positive = values >3 x ULN.

### Relationship of comorbidities with joint count and erythrocyte sedimentation rate

**Fig 1:**
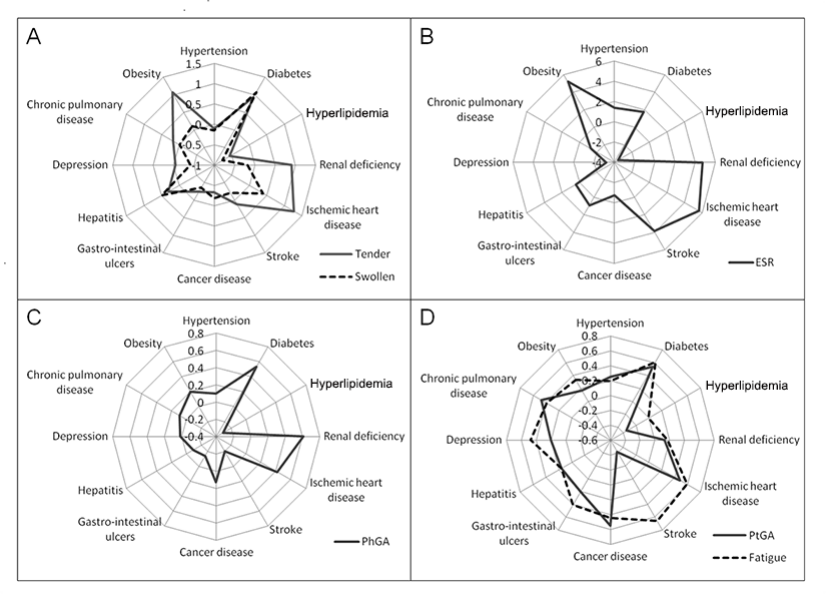
Association between comorbidities and clinical and laboratory outcomes. (A) The number of swollen and tender joints correlates significantly with diabetes and hyperlipidemia; tender joints correlate also with ischemic heart disease and obesity. (B) Erythrocyte sedimentation rate (ESR) correlates significantly with ischemic heart disease, obesity, hyperlipidemia and depression. (C) Physician’s global assessment (PhGA) was significantly associated with diabetes, hyperlipidemia and renal deficiency. (D) Patient’s global assessment (PtGA) correlates significantly with hypertension, diabetes, hyperlipidemia, cancer and chronic pulmonary disease; fatigue was significantly associated with diabetes, ischemic heart disease, gastro-intestinal ulcers, depression, chronic pulmonary disease and obesity.

The number of swollen joints was significantly greater in RA patients with diabetes mellitus than in non-diabetic patients (MD 1.06, 95% CI 0.55 to 1.57) and lower in patients with hyperlipidemia (MD -0.77, 95% CI -1.17 to -0.38). Tender joints number was greater in patients with diabetes (MD 0.93, 95% CI 0.23 to 1.63), ischemic heart disease (MD 1.27, 95% CI 0.21 to 2.34) and obesity (MD 1.07, 95% CI 0.54 to 1.59) and lower in hyperlipidemic patients (MD -0.56, 95% CI -1.10 to -0.02) (Fig 1A).

ESR levels were significantly higher in patients with history of ischemic heart disease (MD 5.64, 95% CI 1.06 to 10.22), in obese patients (MD 5.20, 95% CI 2.93 to 7.47) and lower in hyperlipidemic patients (MD -3.56, 95% CI -5.90 to -1.23) and in patients with history of depression (MD -3.20, 95% CI -6.38 to -0.02) (Fig 1B). No other relationship between comorbidities and joint count or ESR was statistically significant (S4–S6 Tables).

### Relationship of comorbidities with physician’s and patient’s reported outcomes

PhGA was significantly influenced by diabetes (MD 0.53, 95% CI 0.25 to 0.81), hyperlipidemia (MD -0.31, 95% CI -0.52 to -0.09) and renal deficiency (MD 0.61, 95% CI 0.03 to 1.19) (Fig 1C). PtGA was influenced by concomitant hypertension (MD 0.25, 95% CI 0.02 to 0.47), diabetes (MD 0.54, 95% CI 0.21 to 0.86), hyperlipidemia (MD -0.35, 95% CI -0.60 to -0.09), history of cancer disease (MD 0.55, 95% CI 0.08 to 1.03) and chronic pulmonary disease (MD 0.47, 95% CI 0.13 to 0.81). Patient’s evaluation of fatigue was worse with concomitant diabetes (MD 0.61, 95% CI 0.25 to 0.96), ischemic heart disease (MD 0.59, 95% CI 0.05 to 1.13), gastro-intestinal ulcers (MD 0.41, 95% CI 0.09 to 0.73), depression (MD 0.47, 95% CI 0.10 to 0.83), chronic pulmonary disease (MD 0.39, 95% CI 0.02 to 0.77) and obesity (MD 0.33, 95% CI 0.07 to 0.60) (Fig 1D). Complete data about the other analyzed comorbidities are presented in S1–S3 Tables.

### Relationship of comorbidities with DAS28

DAS28 was significantly higher in patients with concomitant diabetes (MD 0.34, 95% CI 0.15 to 0.54), ischemic heart disease (MD 0.40, 95% CI 0.10 to 0.70) and obesity (MD 0.34, 95% CI 0.19 to 0.48) and lower in patients with hyperlipidemia (MD -0.30, 95% CI -0.45 to -0.15) as collected in Table 2.

**Table 2:** Influence of comorbidities on DAS28-ESR.

Comorbidity	Crude MD (95%CI)	MD (95%CI) ^a^	MD (95%CI) ^b^
Hypertension	0.10 (0.00,0.21)	0.11 (-0.01,0.22)	0.06 (-0.08,0.19)
Diabetes	0.43 (0.26,0.59)	**0.44 (0.27,0.61)**	**0.34 (0.15,0.54)**
Hyperlipidemia	-0.20 (-0.32,-0.08)	**-0.20 (-0.33,-0.07)**	**-0.30 (-0.45,-0.15)**
Renal deficiency	0.40 (0.07,0.73)	**0.50 (0.15,0.85)**	0.28 (-0.13,0.68)
Ischemic heart disease	0.17 (-0.07,0.41)	**0.32 (0.08,0.57)**	**0.40 (0.10,0.70)**
Stroke	-0.08 (-0.45,0.30)	-0.04 (-0.45,0.36)	-0.03 (-0.52,0.47)
Cancer disease	-0.18 (-0.41,0.05)	-0.21 (-0.45,0.03)	-0.05 (-0.33,0.24)
Gastro-intestinal ulcers	0.19 (0.03,0.35)	0.07 (-0.09,0.23)	0.02 (-0.16,0.19)
Hepatitis	0.23 (-0.02,0.47)	0.24 (-0.02,0.49)	0.13 (-0.14,0.41)
Depression	0.10 (-0.09,0.29)	-0.05 (-0.24,0.14)	-0.08 (-0.29,0.13)
Chronic pulmonary disease	0.16 (-0.01,0.34)	0.13 (-0.05,0.31)	0.08 (-0.13,0.29)
Obesity	0.35 (0.22,0.48)	**0.36 (0.23,0.49)**	**0.34 (0.19,0.48)**

MD: mean difference; CI: confidence interval; DAS28-ESR: Disease Activity Score using 28-joints count and erythrocyte sedimentation rate. Statistically significant MD (based on confidence interval) in bold.

^a^ adjusted for age, gender, treatments (corticosteroids, NSAIDs, DMARDs), disease duration and serology

^b^ adjusted for age, gender, treatments (corticosteroids, NSAIDs, DMARDs), disease duration, serology and other comorbidities

**Fig 2:**
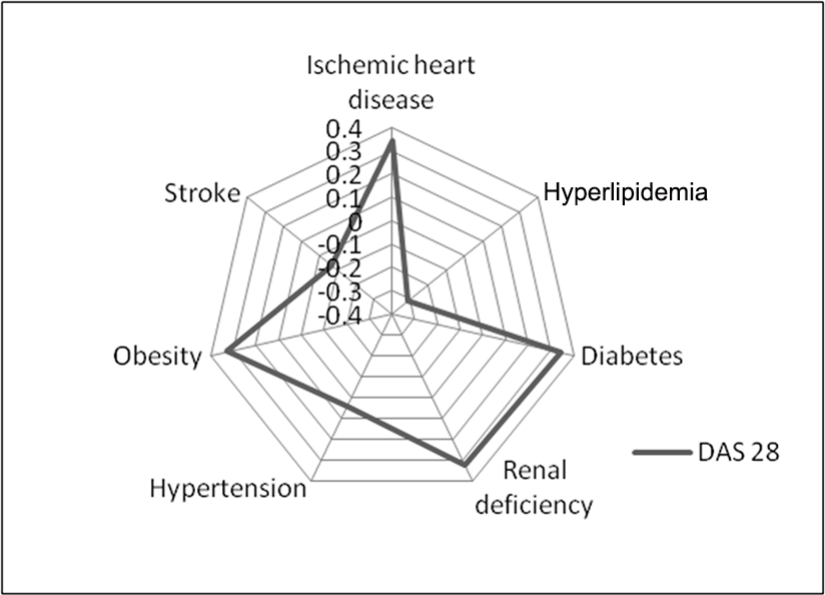
Association between cardiovascular comorbidities and DAS28-ESR. The correlation was statistically significant with concomitant diabetes, hyperlipidemia, ischemic heart disease and obesity. DAS28, Disease Activity Score using 28 joints.

Considering only the cardiovascular comorbidities set the relationship with disease activity corrected for the main confounders and for smoking status is summarized in Table 3 and graphically represented in Fig 2.

**Table 3:** Influence of cardiovascular comorbidities on DAS28-ESR.

Comorbidity	MD (95%CI) ^a^
Hypertension	0.04 (-0.08,0.17)
**Diabetes**	**0.34 (0.15,0.54)**
**Hyperlipidemia**	**-0.31 (-0.44,-0.17)**
Renal deficiency	0.32 (-0.36,0.69)
**Ischemic heart disease**	**0.34 (0.08,0.60)**
Stroke	-0.07 (-0.50,0.34)
**Obesity**	**0.33 (0.19,0.46)**

MD: mean difference; CI: confidence interval; DAS28-ESR: Disease Activity Score using 28-joints count and erythrocyte sedimentation rate. Statistically significant MD (based on confidence interval) in bold.

^a^ adjusted for age, gender, treatments (corticosteroids, NSAIDs, DMARDs), disease duration and serology, smoking status and other comorbidities.

The statistically significant relationship of diabetes, hyperlipidemia, ischemic heart disease and obesity with disease activity was confirmed also by a sub-analysis performed using subgroups of patients stratified according to DAS28, showing that the driver of the association is the subgroup with still active disease (data not shown).

The same analyses have been done for clinical disease activity index (CDAI) with similar results. CDAI was higher in patients with diabetes (MD 3.09, 95% CI 1.60 to 4.57), ischemic heart disease (MD 2.61, 95% CI 0.36 to 4.86) and obesity (MD 1.64, 95% CI 0.53 to 2.74) and lower in patients with hyperlipidemia (MD -2.04, 95% CI -3.19 to -0.90).

### Influence of concomitant corticosteroids and lipid lowering therapy

To test the potential effect of concomitant treatments over these results, exploratory analyses on corticosteroids and statins in patients with specific comorbidities were performed. In diabetic patients, the cumulative dose of corticosteroids was significantly higher compared to non-diabetic patients (MD 1781 mg of equivalent prednisone, 95% CI 106.13 to 3456.66). Patients with hyperlipidemia taking statins had a significantly lower DAS28 compared to patients without hyperlipidemiaor lipid lowering therapy (MD -0.32, 95% CI -0.50 to -0.15), while hyperlipidemic patients not receiving statins and patients receiving statins not being hyperlipidemic did not have significantly lower DAS28 (MD -0.21, 95% CI -0.45 to 0.0.2 and 0.11, 95% CI -0.44 to 0.67, respectively).

## Discussion

The COMORA study represents, to our knowledge, the first attempt to describe the prevalence and relevance of comorbidities on a large cohort of real-life RA patients. The demographic and clinical features of this cohort are comparable to those reported in other RA populations [16–19].

Considering the relationship of each comorbidity with measures of disease activity, after the correction for treatment, disease severity and other concomitant comorbidities, we found that cardiovascular comorbidities more than others correlate with disease activity. In particular hyperlipidemia results associated with lower disease activity, while diabetes, ischemic heart disease and obesity are associated with higher disease activity. Moreover, hyperlipidemia and diabetes seem to be consistently related with disease activity as for all the outcome measure considered, including swollen joint count.

Thirty-two percent of RA patients had an altered lipid profile [14]. We found that these patients had less swollen and tender joints, lower ESR, PtGA and PhGA. It has been previously shown in literature that patients with active RA have lower total cholesterol levels as well as lower high-density lipoprotein (HDL) and low-density lipoprotein (LDL) cholesterol levels compared to healthy controls, while these parameters increase with the institution of anti-inflammatory treatment [20,21]. A number of studies show the effects of conventional DMARDs on lipid profile [22–24]. Concerning biologics, several studies showed considerable increase in serum levels of total cholesterol, HDL and triglycerides in patients receiving either anti-TNF therapy [25,26] or tocilizumab [27,28]. Moreover in responders total cholesterol and HDL increase more compared with non-responders [29]. Therefore, the association of hyperlipidemia with better objective and physician based (swollen joints, ESR and PhGA) and patient’s subjective (tender joints and PtGA) measures of disease activity might be explained by the higher level of lipoproteins in patients presenting with milder disease, receiving effective treatment and with better response. The role of statins in this subgroup has also been investigated: hyperlipidemic patients receiving statins had significantly lower disease activity. This is in keeping with previous studies, showing a better response to treatment in RA patients receiving statins on a DMARD background, with a more evident decrease of acute phase reactants in case of statin administration [30,31].

The prevalence of diabetes in the COMORA was 14% [14]. Patients with diabetes had more swollen and tender joints, worse PtGA and PhGA and worse patient’s evaluation of fatigue. Diabetes mellitus can be related to musculoskeletal comorbidities, such as Charcot arthropathy, adhesive capsulitis, tenosynovitis, Duputryen’s disease and diffuse hyperostosis [32] that may affect patient’s subjective measures of disease activity (tender joints, PtGA, and fatigue). Moreover, in diabetic patients flexor tenosynovitis and osteoarthritis are highly prevalent [33], with a possible influence also on objective measures such as swollen joints and, as a consequence, PhGA [34]. However, diabetic patients had an overall higher prednisone equivalent intake and this might support the hypothesis of a more active disease since presentation, leading to a more aggressive treatment, with diabetes being the potential consequence of longer term and higher dose corticosteroid use. Unfortunately due to the cross-sectional design of the COMORA study, interesting data about the presence of diabetes at baseline are lacking. Moreover hyperglycemia is known to induce IL-1β expression in different immune cells leading to a pro-inflammatory macrophage phenotype proliferation and a persistent inflammatory response [35,36]. IL-1 β, in turn, is the most potent β-cell cytotoxic cytokine [37] leading not only to β-cells inflammation, dysfunction and apoptosis, but may also cause oxidative stress, insulin resistance and inflammation in peripheral tissues [38].

Concerning obesity, it has been recognized that RA associates with altered body composition with an overall increase of body mass index [39]. Classic cachexia with low BMI in RA is associated with severe systemic inflammation, disease activity and increased cardiovascular risk, but it is rarely seen [40]. In the COMORA population 93 patients (2.7%) have a BMI<18 [14] and in the present study we found that results were not affected by the exclusion of these patients (data not shown). In rheumatoid cachexia, with loss of muscle mass and increased fat mass, BMI is usually normal and it has been shown that muscle wasting is associated with higher disease activity [40]. Adipose tissue is also the site of production of adipokines, a class of cytokines involved in the production of several pro-inflammatory factors and acute-phase reactants [41,42].and the role of adipokines in atherosclerosis and cardiovascular risk in RA patients has been already reported [43]. In the COMORA population the mean BMI was 26.1 kg/m^2^ (SD ± 5.5), which is consistent with overweight. In our study obesity–i.e. BMI ≥ 30 kg/m^2^ –was associated with a greater number of tender joints, increased fatigue and higher ESR levels. These results are in keeping with the study by Ajeganova and co-workers in which obese patients had more pain, worse global health and higher disease activity at the time of diagnosis [44].

In our study, patients with history of ischemic heart disease had more tender joints, worse fatigue and higher ESR levels. Considering the strict correlation between disease activity and cardiovascular risk in RA [13], it was not surprising that patients with history of cardiovascular ischemic events had a more active disease. However, a direct influence of the comorbidity itself, especially on patient’s reported measures, cannot be excluded.

Other chronic diseases such as chronic pulmonary diseases, gastro-intestinal ulcers, history of cancer and depression affect patient’s reported outcomes, while renal deficiency seems to affect PhGA, partially related to increased levels of acute phase reactants as a result of a chronic inflammatory state which manifests commonly in end-stage renal failure [45]. Renal deficiency showed a significant relationship with DAS28 in the set of results adjusted for the main confounders but not in the set adjusted also for the presence of other comorbidities. This finding can be explained by the strength association between cardiovascular comorbidities and renal failure.

DAS28, as well as CDAI, results higher in patients with diabetes, obesity and history of ischemic heart disease and lower in hyperlipidemic patients, which was consistent with the relationship of these comorbidities with single measures of disease activity.

The present analysis carries some other limitations: in particular, the cross-sectional design of the COMORA study itself does not allow to evaluate the influence of comorbidities on therapeutic response and to clearly establish a causal relationship between comorbidites, disease characteristics and treatment. Moreover, some painful diseases such as osteoarthritis and extra-articular rheumatisms, in particular fibromyalgia, which may influence disease activity assessment, have not been investigated. Therefore in our study it is not possible to state if clinical composite indexes could be affected by this relevant set of diseases. Finally, some associations could be biased due to different distribution of the comorbidities across country or to the heterogeneity of the relationship between patient-reported and more objective measures of disease activity, as recently demonstrated in the COMORA dataset [46].

## Conclusions

The results of our analyses confirm the relevance of different comorbidities on disease activity in RA. In fact, not only the number but also the type of concomitant diseases should be taken into account in disease activity assessment. In our study, cardiovascular comorbidities in particular correlate significantly with disease activity, with a complex relationship with diabetes and hyperlipidemia. The presence of cardiovascular comorbidities more than others should be considered when deciding on therapeutic strategies in RA patients. Further longitudinal studies are needed in order to confirm these findings, including also other comorbidity patterns, and to investigate the relationship between comorbidities, disease course and response to treatment.

## Supporting Information

S1 Table Influence of comorbidities on swollen joint count. (PDF)

S2 Table Influence of comorbidities on tender joint count. (PDF)

S3 Table Influence of comorbidities on erythrocyte sedimentation rate. (PDF)

S4 Table Influence of comorbidities on physician’s global assessment of disease activity. (PDF)

S5 Table Influence of comorbidities on patient’s global assessment of disease activity. (PDF)

S6 Table Influence of comorbidities on fatigue. (PDF)
