# Supplementary material for: Cardiovascular Comorbidities Relate More than Others with Disease Activity in Rheumatoid Arthritis
Source: PLoS One. 2016 Jan 12;11(1):e0146991. doi: 10.1371/journal.pone.0146991 (PMC4710534; doi:10.1371/journal.pone.0146991)
Supplement: S1 Table — (PDF) [file pone.0146991.s001.pdf]

Table S1. Influence of comorbidities on swollen joint count

Comorbidity	Crude MD (95%CI)	MD (95%CI) [a]	MD (95%CI) [b]
Hypertension	-0.29 (-0.54,-0.03)	-0.27 (-0.56,0.03)	-0.15 (-0.50,0.21)
Diabetes	0.70 (0.29,1.11)	0.74 (0.30,1.18)	1.06 (0.55,1.57)
Hyperlipidemia	-0.7 (-0.97,-0.39)	-0.71 (-1.0,-0.38)	-0.77 (-1.17,-0.38)
Renal deficiency	0.16 (-0.66,0.98)	0.26 (-0.64,1.16)	-0.19 (-1.23,0.86)
Ischemic heart disease	-0.27 (-0.87,0.33)	0.13 (-0.50,0.77)	0.39 (-0.39,1.16)
Stroke	-0.62 (-1.54,0.31)	-0.26 (-1.29,0.77)	-0.21 (-1.46,1.05)
Cancer disease	-0.45 (-1.01,0.12)	-0.56 (-1.18,0.06)	-0.19 (-0.94,0.55)
Gastro-intestinal ulcers	-0.16 (-0.56,0.24)	-0.34 (-0.76,0.07)	-0.36 (-0.82,0.10)
Hepatitis	0.33 (-0.29,0.95)	0.54 (-0.12,1.20)	0.50 (-0.22,1.21)
Depression	-0.20 (-0.66,0.26)	-0.48 (-0.97,0.00)	-0.41 (-0.94,0.11)
Chronic pulmonary disease	0.07 (-0.36,0.49)	-0.08 (-0.54,0.39)	-0.01 (-0.55,0.53)
Obesity	0.06 (-0.26,0.39)	-0.05 (-0.39,0.29)	0.10 (-0.28,0.48)

MD: mean difference; CI: confidence interval

[a] adjusted for age, gender, treatments (corticosteroids, NSAIDs, DMARDs), disease duration and serology

[b] adjusted for age, gender, treatments (corticosteroids, NSAIDs, DMARDs), disease duration, serology and other comorbidities
