# Supplementary material for: Cardiovascular Comorbidities Relate More than Others with Disease Activity in Rheumatoid Arthritis
Source: PLoS One. 2016 Jan 12;11(1):e0146991. doi: 10.1371/journal.pone.0146991 (PMC4710534; doi:10.1371/journal.pone.0146991)
Supplement: S2 Table — (PDF) [file pone.0146991.s002.pdf]

Table S2. Influence of comorbidities on tender joint count

Comorbidity	Crude MD (95%CI)	MD (95%CI) [a]	MD (95%CI) [b]
Hypertension	-0.08 (-0.43,0.27)	0.03 (-0.38,0.43)	-0.11 (-0.60,0.37)
Diabetes	1.10 (0.55,1.65)	1.12 (0.52,1.72)	0.93 (0.23,1.63)
Hyperlipidemia	-0.38 (-0.78,0.02)	-0.34 (-0.79,0.11)	-0.56 (-1.10,-0.02)
Renal deficiency	1.01 (-0.10,2.12)	1.61 (0.39,2.84)	0.90 (-0.53,2.32)
Ischemic heart disease	0.30 (-0.51,1.12)	0.96 (0.10,1.82)	1.27 (0.21,2.34)
Stroke	-0.21 (-1.47,1.05)	-0.07 (-1.48,1.35)	0.12 (-1.61,1.84)
Cancer disease	-0.62 (-1.39,0.15)	-0.72 (-1.57,0.12)	-0.33 (-1.34,0.69)
Gastro-intestinal ulcers	0.33 (-0.21,0.87)	-0.01 (-0.58,0.56)	-0.25 (-0.88,0.38)
Hepatitis	0.52 (-0.32,1.37)	0.73 (-0.18,1.64)	0.31 (-0.67,1.29)
Depression	0.36 (-0.26,0.98)	0.03 (-0.63,0.69)	-0.04 (-0.76,0.69)
Chronic pulmonary disease	0.57 (0.00,1.14)	0.34 (-0.29,0.97)	0.17 (-0.57,0.90)
Obesity	1.08 (0.64,1.52)	0.92 (0.46,1.38)	1.07 (0.54,1.59)

MD: mean difference; CI: confidence interval

[a] adjusted for age, gender, treatments (corticosteroids, NSAIDs, DMARDs), disease duration and serology

[b] adjusted for age, gender, treatments (corticosteroids, NSAIDs, DMARDs), disease duration, serology and other comorbidities
