# Supplementary material for: Cardiovascular Comorbidities Relate More than Others with Disease Activity in Rheumatoid Arthritis
Source: PLoS One. 2016 Jan 12;11(1):e0146991. doi: 10.1371/journal.pone.0146991 (PMC4710534; doi:10.1371/journal.pone.0146991)
Supplement: S3 Table — (PDF) [file pone.0146991.s003.pdf]

Table S3. Influence of comorbidities on erythrocyte sedimentation rate

Comorbidity	Crude MD (95%CI)	MD (95%CI) [a]	MD (95%CI) [b]
Hypertension	2.76 (1.26,4.27)	1.94 (0.18,3.69)	1.40 (-0.70,3.51)
Diabetes	5.10 (2.70,7.50)	4.21 (1.63,6.79)	1.75 (-1.29,4.80)
Hyperlipidemia	-1.30 (-3.02,0.42)	-2.28 (-4.21,-0.36)	-3.56 (-5.90,-1.23)
Renal deficiency	9.48 (4.65,14.31)	7.52 (2.21,12.83)	4.71 (-1.49,10.90)
Ischemic heart disease	4.50 (1.0,8.0)	4.75 (1.03,8.47)	5.64 (1.06,10.22)
Stroke	1.60 (-3.84,7.03)	3.37 (-2.71,9.45)	3.85 (-3.62,11.32)
Cancer disease	-1.69 (-5.06,1.67)	-2.66 (-6.32,1.0)	-0.74 (-5.15,3.68)
Gastro-intestinal ulcers	2.96 (0.63,5.29)	1.86 (-0.56,4.29)	0.92 (-1.78,3.63)
Hepatitis	1.51 (-2.09,5.11)	1.0 (-2.87,4.9)	0.41 (-3.79,4.60)
Depression	-0.94 (-3.66,1.79)	-3.15 (-6.03,-0.28)	-3.20 (-6.38,-0.02)
Chronic pulmonary disease	-0.53 (-3.03,1.97)	-1.45 (-4.18,1.28)	-1.27 (-4.48,1.93)
Obesity	4.70 (2.82,6.58)	5.36 (3.39,7.33)	5.20 (2.93,7.47)

MD: mean difference; CI: confidence interval

[a] adjusted for age, gender, treatments (corticosteroids, NSAIDs, DMARDs), disease duration and serology

[b] adjusted for age, gender, treatments (corticosteroids, NSAIDs, DMARDs), disease duration, serology and other comorbidities
