# Supplementary material for: Cardiovascular Comorbidities Relate More than Others with Disease Activity in Rheumatoid Arthritis
Source: PLoS One. 2016 Jan 12;11(1):e0146991. doi: 10.1371/journal.pone.0146991 (PMC4710534; doi:10.1371/journal.pone.0146991)
Supplement: S4 Table — (PDF) [file pone.0146991.s004.pdf]

Table S4. Influence of comorbidities on physician's global assessment of disease activity

Comorbidity	Crude MD (95%CI)	MD (95%CI) [a]	MD (95%CI) [b]
Hypertension	0.10 (-0.05,0.25)	0.14 (-0.02,0.31)	0.10 (-0.10,0.29)
Diabetes	0.61 (0.37,0.85)	0.60 (0.36,0.84)	0.53 (0.25,0.81)
Hyperlipidemia	-0.27 (-0.44,-0.10)	-0.14 (-0.32,0.04)	-0.31 (-0.52,-0.09)
Renal deficiency	0.64 (0.17,1.12)	0.83 (0.33,1.32)	0.61 (0.03,1.19)
Ischemic heart disease	0.18 (-0.17,0.53)	0.39 (0.04,0.74)	0.42 (-0.01,0.84)
Stroke	-0.20 (-0.76,0.36)	-0.23 (-0.80,0.34)	-0.20 (-0.89,0.49)
Cancer disease	-0.16 (-0.49,0.17)	-0.08 (-0.43,0.26)	0.13 (-0.28,0.54)
Gastro-intestinal ulcers	0.12 (-0.11,0.35)	-0.06 (-0.29,0.17)	-0.14 (-0.39,0.12)
Hepatitis	0.12 (-0.23,0.47)	0.05 (-0.32,0.41)	-0.09 (-0.48,0.30)
Depression	0.17 (-0.09,0.43)	0.06 (-0.21,0.32)	0.02 (-0.28,0.31)
Chronic pulmonary disease	0.09 (-0.15,0.34)	0.09 (-0.17,0.34)	0.09 (-0.21,0.38)
Obesity	0.27 (0.09,0.45)	0.26 (0.07,0.45)	0.20 (-0.01,0.41)

MD: mean difference; CI: confidence interval

[a] adjusted for age, gender, treatments (corticosteroids, NSAIDs, DMARDs), disease duration and serology

[b] adjusted for age, gender, treatments (corticosteroids, NSAIDs, DMARDs), disease duration, serology and other comorbidities
