# Supplementary material for: Cardiovascular Comorbidities Relate More than Others with Disease Activity in Rheumatoid Arthritis
Source: PLoS One. 2016 Jan 12;11(1):e0146991. doi: 10.1371/journal.pone.0146991 (PMC4710534; doi:10.1371/journal.pone.0146991)
Supplement: S5 Table — (PDF) [file pone.0146991.s005.pdf]

Table S5. Influence of comorbidities on patient's global assessment of disease activity

Comorbidity	Crude MD (95%CI)	MD (95%CI) [a]	MD (95%CI) [b]
Hypertension	0.30 (0.13,0.46)	0.33 (0.14,0.52)	0.25 (0.02,0.47)
Diabetes	0.53 (0.27,0.80)	0.66 (0.38,0.94)	0.54 (0.21,0.86)
Hyperlipidemia	-0.27 (-0.46,-0.08)	-0.12 (-0.33,0.09)	-0.35 (-0.60,-0.09)
Renal deficiency	0.54 (0.01,1.07)	0.50 (-0.08,1.07)	0.13 (-0.54,0.79)
Ischemic heart disease	0.42 (0.03,0.81)	0.55 (0.14,0.96)	0.48 (-0.02,0.98)
Stroke	0.12 (-0.49,0.72)	-0.17 (-0.84,0.50)	-0.42 (-1.23,0.39)
Cancer disease	0.14 (-0.23,0.51)	0.19 (-0.21,0.59)	0.55 (0.08,1.03)
Gastro-intestinal ulcers	0.54 (0.28,0.80)	0.28 (0.02,0.55)	0.20 (-0.09,0.49)
Hepatitis	0.35 (-0.05,0.74)	0.24 (-0.18,0.66)	0.16 (-0.30,0.62)
Depression	0.44 (0.15,0.74)	0.33 (0.02,0.64)	0.20 (-0.14,0.54)
Chronic pulmonary disease	0.41 (0.14,0.68)	0.46 (0.16,0.75)	0.47 (0.13,0.82)
Obesity	0.31 (0.11,0.52)	0.33 (0.12,0.55)	0.17 (-0.08,0.41)

MD: mean difference; CI: confidence interval

[a] adjusted for age, gender, treatments (corticosteroids, NSAIDs, DMARDs), disease duration and serology

[b] adjusted for age, gender, treatments (corticosteroids, NSAIDs, DMARDs), disease duration, serology and other comorbidities
