# Supplementary material for: Cardiovascular Comorbidities Relate More than Others with Disease Activity in Rheumatoid Arthritis
Source: PLoS One. 2016 Jan 12;11(1):e0146991. doi: 10.1371/journal.pone.0146991 (PMC4710534; doi:10.1371/journal.pone.0146991)
Supplement: S6 Table — (PDF) [file pone.0146991.s006.pdf]

Table S6. Influence of comorbidities on fatigue

Comorbidity	Crude MD (95%CI)	MD (95%CI) [a]	MD (95%CI) [b]
Hypertension	0.21 (0.03,0.39)	0.39 (0.18,0.60)	0.19 (-0.06,0.44)
Diabetes	0.62 (0.33,0.91)	0.78 (0.47,1.09)	0.61 (0.25,0.96)
Hyperlipidemia	-0.11 (-0.32,0.10)	0.22 (-0.02,0.45)	-0.01 (-0.29,0.26)
Renal deficiency	0.83 (0.26,1.41)	0.92 (0.29,1.55)	0.17 (-0.54,0.89)
Ischemic heart disease	0.62 (0.20,1.04)	0.74 (0.29,1.19)	0.59 (0.05,1.13)
Stroke	1.25 (0.59,1.91)	1.22 (0.48,1.96)	0.66 (-0.21,1.54)
Cancer disease	0.01 (-0.39,0.41)	0.05 (-0.39,0.49)	0.45 (-0.06,0.97)
Gastro-intestinal ulcers	0.72 (0.44,1.0)	0.53 (0.23,0.82)	0.41 (0.09,0.73)
Hepatitis	0.35 (-0.09,0.78)	0.34 (-0.13,0.80)	0.16 (-0.33,0.66)
Depression	0.73 (0.41,1.05)	0.73 (0.39,1.07)	0.47 (0.10,0.83)
Chronic pulmonary disease	0.52 (0.22,0.81)	0.50 (0.18,0.83)	0.39 (0.02,0.77)
Obesity	0.45 (0.22,0.67)	0.52 (0.28,0.75)	0.33 (0.07,0.60)

MD: mean difference; CI: confidence interval

[a] adjusted for age, gender, treatments (corticosteroids, NSAIDs, DMARDs), disease duration and serology

[b] adjusted for age, gender, treatments (corticosteroids, NSAIDs, DMARDs), disease duration, serology and other comorbidities
